# The diguanylate cyclase AdrA regulates flagellar biosynthesis in *Pseudomonas fluorescens* F113 through SadB

**DOI:** 10.1038/s41598-019-44554-z

**Published:** 2019-05-30

**Authors:** Candela Muriel, Esther Blanco-Romero, Eleftheria Trampari, Eva Arrebola, David Durán, Miguel Redondo-Nieto, Jacob G. Malone, Marta Martín, Rafael Rivilla

**Affiliations:** 1 Departamento de Biología, Universidad Autónoma de Madrid, Campus de Cantoblanco, 28049 Madrid, Spain; 2 Department of Molecular Microbiology, John Innes Centre. Colney Lane, Norwich, UK; 3 Present Address: Quadram Institute, Norwich, UK; 4 Present Address: Department of Microbiology, University of Málaga, Málaga, Spain

**Keywords:** Genetics, Microbiology

## Abstract

Flagellum mediated motility is an essential trait for rhizosphere colonization by pseudomonads. Flagella synthesis is a complex and energetically expensive process that is tightly regulated. In *Pseudomonas fluorescens*, the regulatory cascade starts with the master regulatory protein FleQ that is in turn regulated by environmental signals through the Gac/Rsm and SadB pathways, which converge in the sigma factor AlgU. AlgU is required for the expression of *amrZ*, encoding a FleQ repressor. AmrZ itself has been shown to modulate c-di-GMP levels through the control of many genes encoding enzymes implicated in c-di-GMP turnover. This cyclic nucleotide regulates flagellar function and besides, the master regulator of the flagellar synthesis signaling pathway, FleQ, has been shown to bind c-di-GMP. Here we show that AdrA, a diguanylate cyclase regulated by AmrZ participates in this signaling pathway. Epistasis analysis has shown that AdrA acts upstream of SadB, linking SadB with environmental signaling. We also show that SadB binds c-di-GMP with higher affinity than FleQ and propose that c-di-GMP produced by AdrA modulates flagella synthesis through SadB.

## Introduction

The pseudomonads are motile bacteria able to swim and swarm by means of polar flagella. Flagella are also used in the initial attachment of bacteria to surfaces^1^, and are therefore important for biofilm formation^2^. Flagellar motility is an important trait for rhizosphere colonization^3–5^. In the biocontrol model bacterium *Pseudomonas fluorescens* F113, it has been shown that hypermotile variants arise during rhizosphere colonization^6^ and that this trait is more important than the ability to form biofilms for the competitive colonization of the rhizosphere^7^.

Biosynthesis of the flagellar apparatus is an energetically expensive process that requires the expression of many genes and therefore, it is tightly controlled. A regulatory cascade initiated by the master regulator FleQ and the sigma factor FliA results in the ordered production and assemblage of the flagellar components resulting in a functional flagellum^8,9^. However, the initiation of this regulatory cascade is also affected by environmental cues and as-yet unknown signals. We have previously shown that in *P. fluorescens* F113 two signal transduction pathways, one initiated by SadB and the other by the GacA/GacS two component system converge in the production of the AlgU sigma factor^10^. This sigma factor is required for the expression of the *amrZ* gene, which encodes a global and bifunctional transcriptional regulator, implicated in the expression of hundreds of genes both in *P. fluorescens* F113^11^ and *Pseudomonas aeruginosa*^12^. AmrZ is a strong transcriptional repressor of the gene encoding the flagellar master regulator FleQ in both strains^10,13^.

Bis-(3′-5′)-cyclic dimeric guanosine monophosphate (c-di-GMP) is also an important player in processes related to motility and biofilm formation^14^. Low c-di-GMP levels are associated with high motility and a planktonic life-style, while high levels are associated with the production of exopolysaccharides and biofilm formation and therefore, with a sessile life-style^15^. Intracellular levels of c-di-GMP are the result of the action of two types of enzymes, diguanylate cyclases (DGCs) and phosphodiesterases (PDEs), which carry out the synthesis and degradation of this molecule respectively^16^. DGCs are proteins that contain GGDEF domains^17^, while PDEs either contain EAL^15^ or HD-GYP^18^ domains.

c-di-GMP has been implicated in the control of flagellar function in pseudomonads and other bacteria, including the energization of the apparatus through the FliI ATPase^19^, control of the rotation speed^20,21^ and the reversal frequency^22^. However, the implications of c-di-GMP for flagellar biosynthesis has been less investigated. We have recently shown that AmrZ is a major determinant of c-di-GMP levels in *P. fluorescens* F113 by controlling the transcription of multiple genes encoding DGCs, PDEs and c-di-GMP sensing proteins^23^. The master regulator FleQ^24^. has been shown to bind c-di-GMP and binding of this cyclic dinucleotide to FleQ represses flagella synthesis^25^. The aim of this work was to study the possible role of c-di-GMP in the biogenesis of the flagellar apparatus, as well as to find novel proteins involved in its production and sensing that affect this process in *P. fluorescens* F113.

## Results

### AdrA is a membrane associated diguanylate cyclase

**Figure 1:**
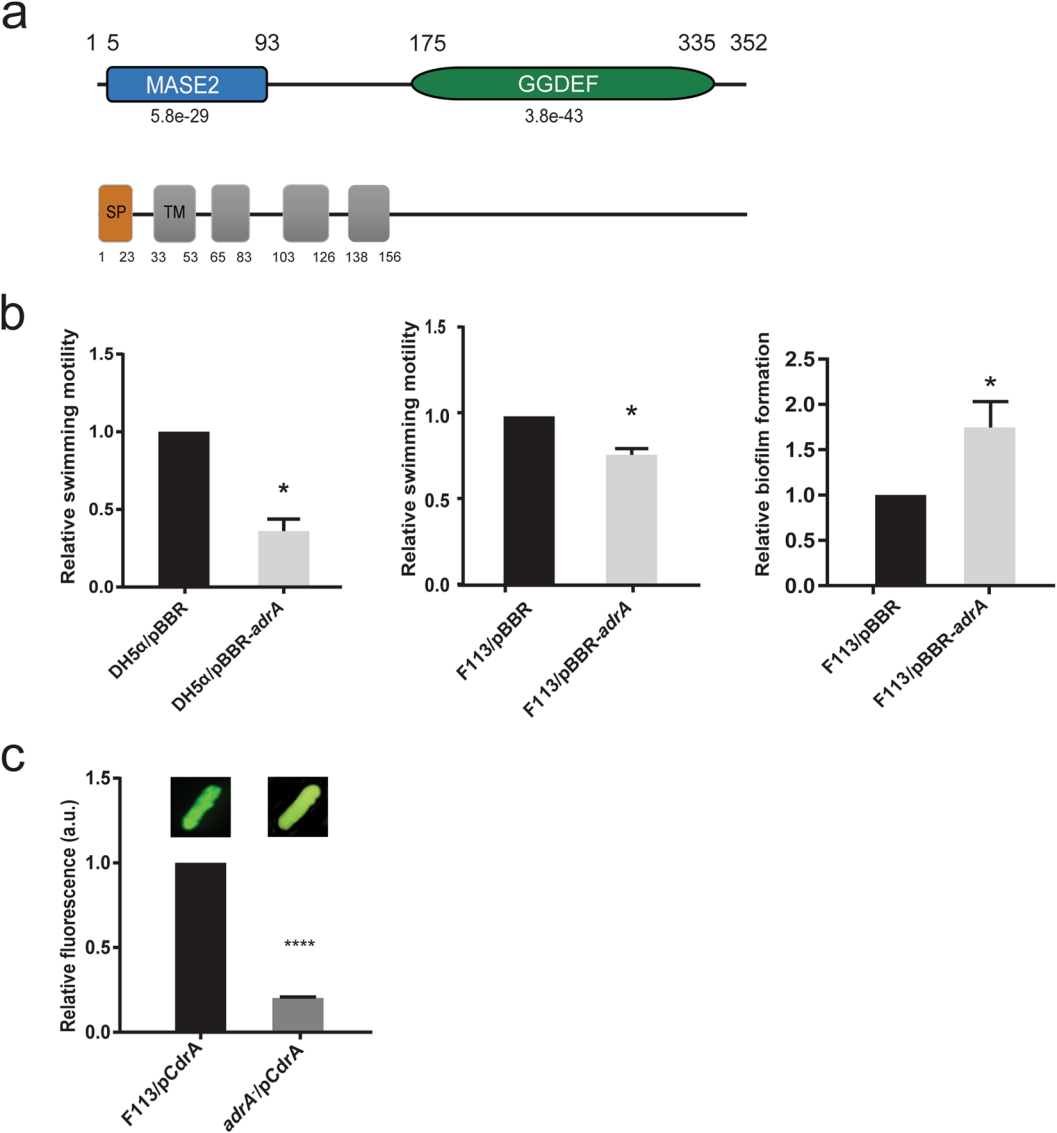
AdrA encoded by PSF113_1982 in *Pseudomonas fluorescens* F113 is a DGC. (**a**) Predicted domain organization for AdrA protein (352 aa) from *P. fluorescens* F113 according to HMMER using profile hidden Markov models and Pfam database. Domains are indicated in each block. Domains (MASE2 and GGDEF) and their individual E-values are shown above. Signal peptide (SP, brown rectangle) and transmembrane domains (TM, grey rectangles) are shown below. Numbers indicate the start and end aa positions covered by each domain or feature. (**b**) Relative swimming motility in DH5α and *P. fluorescens* F113 and relative biofilm formation in *P. fluorescens* F113 in AdrA overexpression experiments. pBBRMCS-5 vector harbouring *adrA* from *P. fluorescens* F113 was used for overexpression experiments in both strains. The empty pBBRMCS-5 vector was used as control. Mean ± SD of three replicates are shown. Statistically significant difference (p < 0.05) are denoted by asterisks. (**c**) AdrA participates in the synthesis of the second messenger c-di-GMP. Streaks on LB medium of *P. fluorescens* F113 and its *adrA* mutant harbouring the *gfp*-based pCdra biosensor for c-di-GMP. Pictures were obtained in a Leika binocular microscope with a GFP filter set and 50 miliseconds of exposition time. Intracelullar levels of c-di-GMP were measured as fluorescence emission in the pCdra-containing strains. Mean ± SD of five analyzed extracts per strain are represented. Asterisks denote statistical significance of the data (****p < 0.0001).

Sequence analysis by HMMER^26^ using profile hidden Markov models and the Pfam database showed that AdrA (PSF113_1982) is a membrane associated protein with an N-terminal extracellular MASE2 domain^27^ and a C-terminal, cytoplasmic GGDEF domain (Fig. 1a). The MASE2 domain contains four predicted transmembrane helices (Fig. 1a) that would link the protein to the cytoplasmic membrane, forming a receptor for as-yet unknown signals. We have previously shown^23^ that an *adrA*^*−*^ mutant in *P. fluorescens* F113 shows increased motility (Supplementary Fig. 1) and a reduction in its attachment to surfaces. In order to confirm the diguanylate cyclase activity of the GGDEF domain, *adrA* was ectopically expressed in *E. coli* DH5α and *P. fluorescens* F113. As shown in Fig. 1b, overexpression of *adrA* resulted in a decrease in swimming motility and an increase in the attachment to surfaces, which is consistent with an increase in c-di-GMP caused by overexpression of a DGC. Moreover, c-di-GMP intracellular measurements in the *adrA* mutant reveal very low levels of this second messenger in the mutant in comparison with the wild-type strain as shown in Fig. 1c. Taken together, these results indicate that the membrane bound AdrA protein possesses DGC activity.

### AdrA regulates flagella synthesis

**Figure 2:**
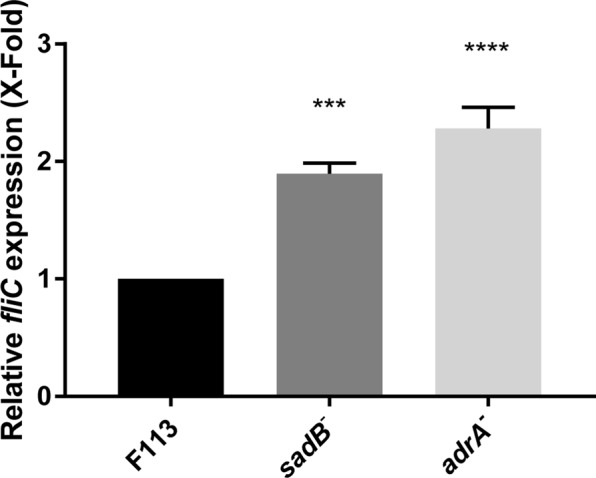
AdrA regulates the expression of the flagellar gene *fliC*. RT-qPCR analysis of *fliC* expression in *Pseudomonas fluorescens* F113 and its *adrA* and *sadB* mutant backgrounds. Gene expression was normalized with *rpoZ* and relativized to wt. Mean ± SD of three replicates are shown. Asterisks indicate statistically significant differences (***p < 0.001, ****p < 0.0001).

In order to determine whether AdrA participates in the regulation of flagella synthesis, we tested the expression of the *fliC* gene in *P. fluorescens* F113 and its *adrA* mutant background. As shown in Fig. 2, expression of *fliC* is significantly higher in the *adrA* mutant. SadB is a signal transduction protein that has been shown to regulate flagella synthesis in F113^10,28^. Consistent with this, similar results were obtained in a *sadB*^*−*^ background. Together, these results show that AdrA and SadB participate in the regulation of flagellar gene expression and therefore in flagella synthesis.

### AdrA contributes to the SadB signaling pathway but acts independently of the GacAS system

**Figure 3:**
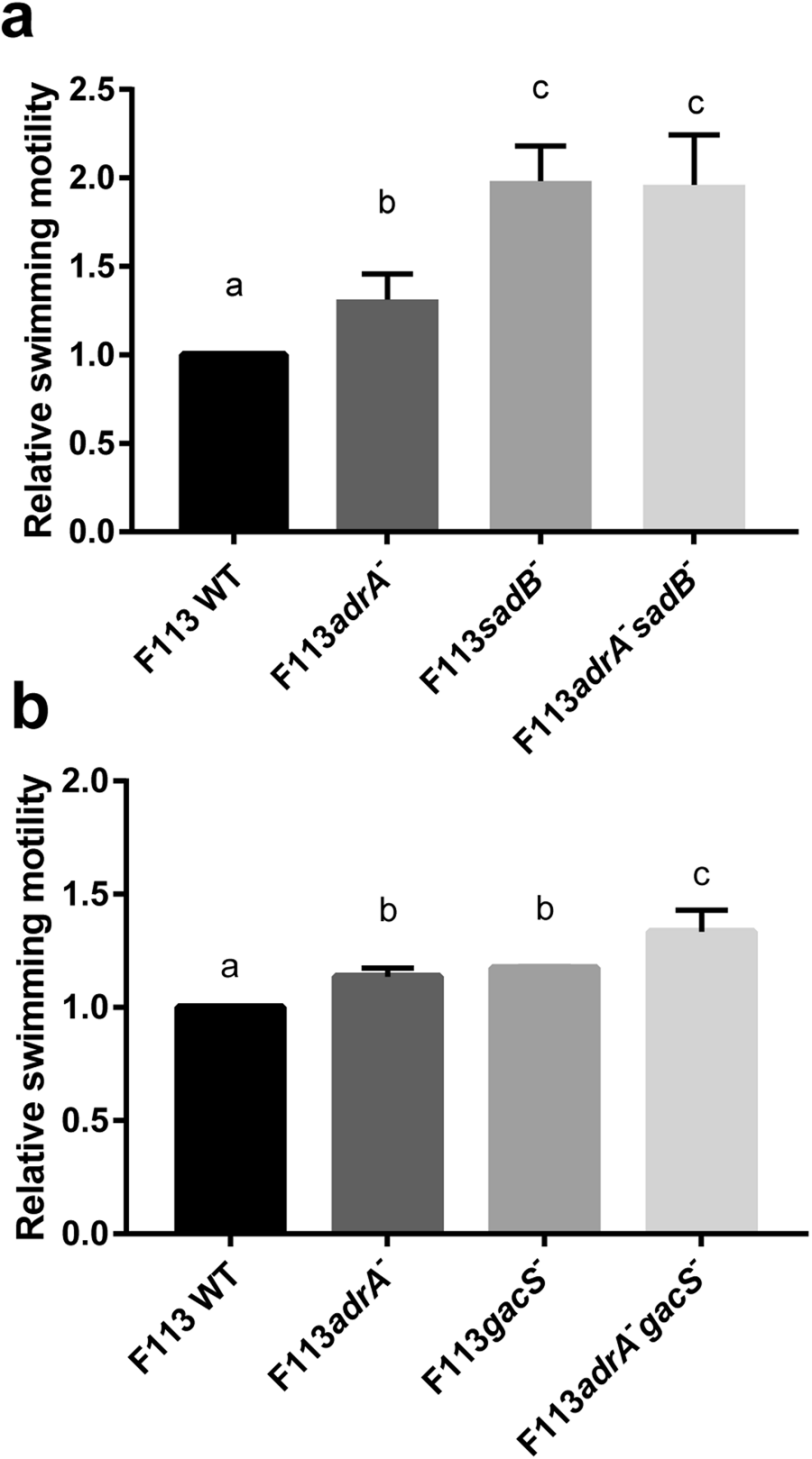
AdrA regulates flagellar synthesis through SadB and independently of Gac/Rsm pathway in *Pseudomonas fluorescens* F113. Relative swimming motility of *P. fluorescens* F113, simple or double mutants affected in *adrA* and *sadB* (**a**) or *gacS* (**b**) genes. Mean ± SD of three replicates are shown. Different letters indicate statistically significant differences (p < 0.05).

In order to investigate the participation of AdrA in the flagella biosynthesis pathway, we constructed a double mutant in the *adrA* and *sadB* genes. As shown in Fig. 3a, the *sadB* mutant showed a hypermotility phenotype stronger than the *adrA* mutant. The double mutant showed a non-additive phenotype, indicating genetic interaction between *adrA* and *sadB*. Furthermore, the double mutant showed a phenotype identical to the phenotype of the *sadB* mutant, suggesting that AdrA acts upstream of SadB in the flagella synthesis regulatory pathway. We also tested the possible interaction of *adrA* with *gacS*, a gene that acts in the flagella synthesis regulatory pathway independently of *sadB*^10^. As shown in Fig. 3b, the *adrAgacS* double mutant showed an additive phenotype in comparison with the swimming motility pathways of the individual mutants. These results indicate that AdrA and GacS act independently in the regulation of flagella synthesis in *P. fluorescens* F113.

### SadB binds c-di-GMP

**Figure 4:**
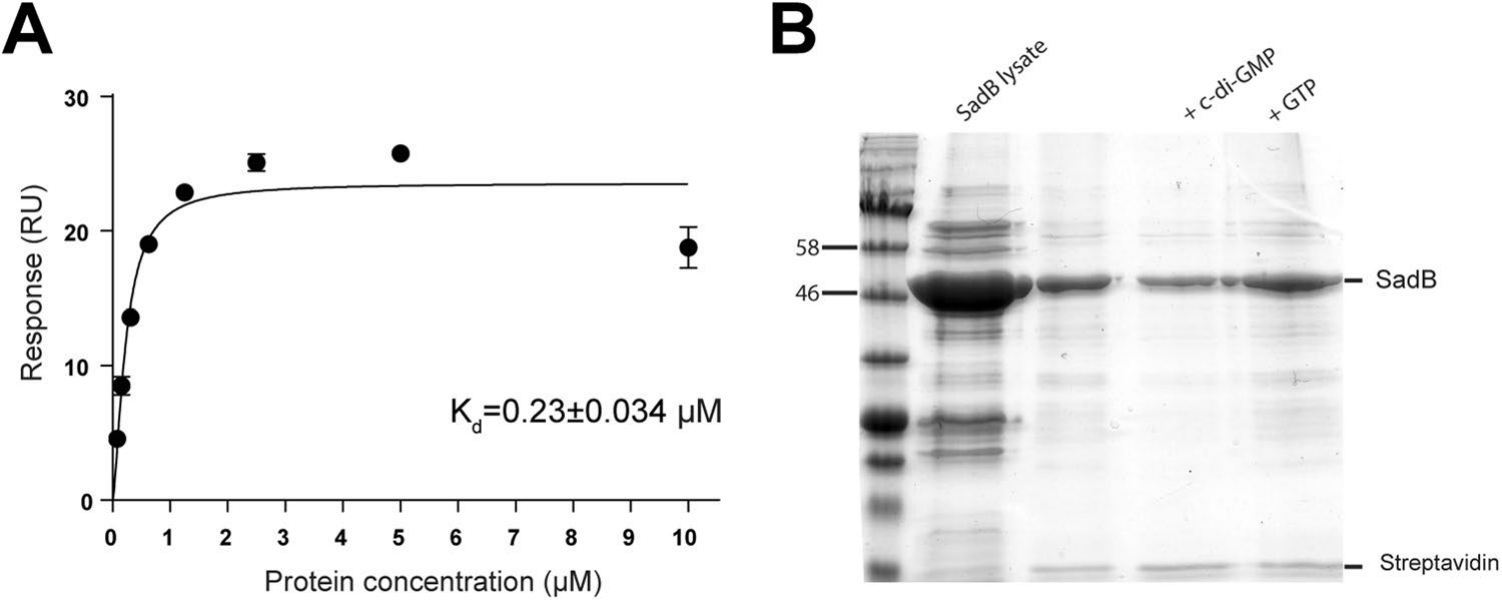
SadB is a c-di-GMP binding protein in *Pseudomonas fluorescens* F113. (**A**) SPR affinity fit curve describing SadB-c-di-GMP binding. (**B**) Streptavidin UV Precipitation (SUPr) assay. Biotinylated c-di-GMP was used for the precipitation of SadB from an induced lysate. Free c-di-GMP and GTP were used as competitors.

The SadB protein contains two domains of unknown function. The N-terminal YbaK-like domain has been suggested to bind nucleotides or oligonucleotides^29^. Furthermore, the C-terminal HDOD domain resembles the PDE HD-GYP domain. However, the HDOD domain does not possess catalytic activity and it has been suggested that it might bind c-di-GMP^30^. Considering the presence of these two domains and the AdrA DGC activity upstream of SadB, we decided to test whether SadB binds c-di-GMP. N-tagged HA-SadB protein was produced and gel filtration showed an apparent MW compatible with a dimeric conformation both in the presence or absence of c-di-GMP (data not shown). Surface Plasmon Resonance (SPR) analysis of HA-SadB in the presence of c-di-GMP showed that the protein was able to bind the cyclic nucleotide with a dissociation constant (K_d_) of 0.23 µM (Fig. 4A). The specificity of the binding was then confirmed using a pull-down assay with biotinylated c-di-GMP (Fig. 4B), which shows that streptavidin precipitation of the SadB protein could be disrupted by addition of free c-di-GMP but not by GTP.

## Discussion

Biogenesis of the flagellar apparatus in pseudomonads is activated by the master regulator FleQ, which initiates a regulatory cascade^8^. In *Pseudomonas fluorescens* F113^10^ and in *P. aeruginosa*^12^, the expression of the *fleQ* gene is transcriptionally repressed by AmrZ, a bifunctional regulator whose expression depends on the sigma factor AlgU^13^. Additionally, a reciprocal transcriptional repression between FleQ and AmrZ has been shown in *P. fluorescens* F113^31^. In this strain, expression of *algU* is under the control of two convergent pathways: the Gac/Rsm pathway and the SadB pathway^10^.

The role of c-di-GMP in the regulation of flagellar synthesis is evident at different levels. First, it has been shown that FleQ is a c-di-GMP binding protein^24^ that in response to cyclic nucleotide binding functions either as a transcriptional activator or as a repressor^32^. Thus, high levels of c-di-GMP repress flagella synthesis through FleQ^25^, activating in turn genes for the production of exopolysaccharides related to biofilm formation^32^. Here we show that the SadB protein, a regulator involved in the control of flagella synthesis that acts upstream of FleQ, specifically binds c-di-GMP at physiological levels and with an affinity that is much higher than that of FleQ. Furthermore, another regulatory protein, AmrZ, has been shown to be a major determinant of c-di-GMP levels in *P. fluorescens* F113. In this bacterium, AmrZ acts as a transcriptional activator of genes encoding DGCs and other proteins associated with c-di-GMP turnover^23^.

Results presented here show that at least one AmrZ regulated DGC, AdrA, encoded by PSF113_1982 participates in the regulatory pathway resulting in flagella biogenesis. AdrA is a transmembrane protein with a conserved C-terminal GGDEF cytoplasmic domain and an N-terminal MASE2 integral membrane sensory domain. MASE2 domains have unknown function but are often found adjacent to GGDEF domains in bacterial signaling proteins^27^ (Fig. 1a). Although sequence homology is limited, the domain architecture of AdrA is identical to the AdrA protein in *Salmonella typhimurium*^33^ and its *E. coli* orthologue YaiC. Furthermore, the same transmembrane helices are present in the MASE2 domain. The signal detected by MASE2 domains is unknown, but this domain is often found associated with nucleotide cycling domains in DGCs and adenylate cyclases^34^. In *E. coli* and *S. typhimurium*, AdrA is a diguanylate cyclase implicated in c-di-GMP-mediated cellulose production and biofilm formation^35^. Results obtained in this study (Fig. 1b) indicate that in F113, AdrA could also act as a DGC. AdrA proteins are present in most strains of the *Pseudomonas fluorescens* group, one of the main clusters within the *P. fluorescens* complex of species^36,37^. Besides this group, AdrA orthologues are present in the genomes of *P. syringae*, *P. putida* and *P. stutzeri*, but absent in *P. aeruginosa*. To our knowledge, its relevance in swimming motility and/or biofilm formation has not been explored in strains other than *P. fluorescens* F113. Previous analysis showed that in *P. fluorescens* F113, AdrA is involved in swimming and biofilm formation, since inactivation of the *adrA* gene resulted in increased swimming motility and a reduction in the initial stages of attachment to surfaces^23^. Furthermore, to confirm that AdrA acts as a DGC we have carried out the overexpression of this gene in *E. coli* DH5α and F113, resulting in a substantial decrease in swimming motility comparable to the effect described for the DGC SadC (Fig. 1b)^20^. Results show its implication in flagellar gene expression, since an *adrA* mutant shows enhanced *fliC* expression. Additionally, genetic interaction between *adrA* and *sadB* indicates that, AdrA participates in the regulation of flagella biogenesis. The swimming motility phenotype of the *sadB* mutation is dominant, suggesting that AdrA might act upstream of SadB. Since AdrA is a predicted membrane protein with a putative sensory domain, this protein links SadB signaling with possible environmental signals. Consequently, our data suggest that AdrA acts upstream of SadB, in the regulatory cascade. Moreover, our data indicate that AdrA acts independently of the Gac system, which is also consistent with the activity of AdrA in the SadB branch of the signaling pathway^10^.

SadB has been described as a signal transduction protein that negatively regulates motility in *P. aeruginosa*^38^ and *P. fluorescens*^28^. SadB contains two domains that might be implicated in c-di-GMP binding, a HDOD domain and an YbaK like domain. The HDOD domain resembles the HD-GYP domain but lacks its characteristic c-di-GMP phosphodiesterase activity^39^. Proteins with HDOD domains have been studied in *Xanthomonas campestris*. The HdpA protein from this bacterium has been shown to be implicated in bacterial attachment^40^, while the GsmR protein has been confirmed to affect the transcription of genes involved in flagella synthesis, including *fliC*^41^. It is therefore likely that HDOD proteins play similar roles in *X. campestris* and *P. fluorescens*. However, the mechanistic role (s) of HDOD domain has not been established. The similarity of the HDOD domain with the HD-GYP domain and its lack of phosphodiesterase activity prompted Merritt and coworkers and Navazo and coworkers^28,30^ to suggest that the HDOD domain in SadB could bind c-di-GMP. Regarding the YbaK-like domain, which resembles the active sites of deacylases, its function is unknown. However, it seems to be important as insertions in either of the two domains abolish SadB function in *P. aeruginosa*^42^. In *Haemophilus influenzae*, structural analysis of its YbaK-containing protein has shown the presence of a putative binding site that might accommodate a nucleotide or oligonucleotide^29^. We have shown here that the SadB protein is able to bind c-di-GMP specifically and with high affinity. Since binding of c-di-GMP to FleQ protein and the implication of this messenger in the transcription of flagellar genes in pseudomonads has been already reported^24,32^, our finding establishes another checkpoint for c-di-GMP regulation, upstream in the pathway. It is interesting to note that the apparent affinity of SadB for c-di-GMP is about 30 times higher than the reported for FleQ^25^.

**Figure 5:**
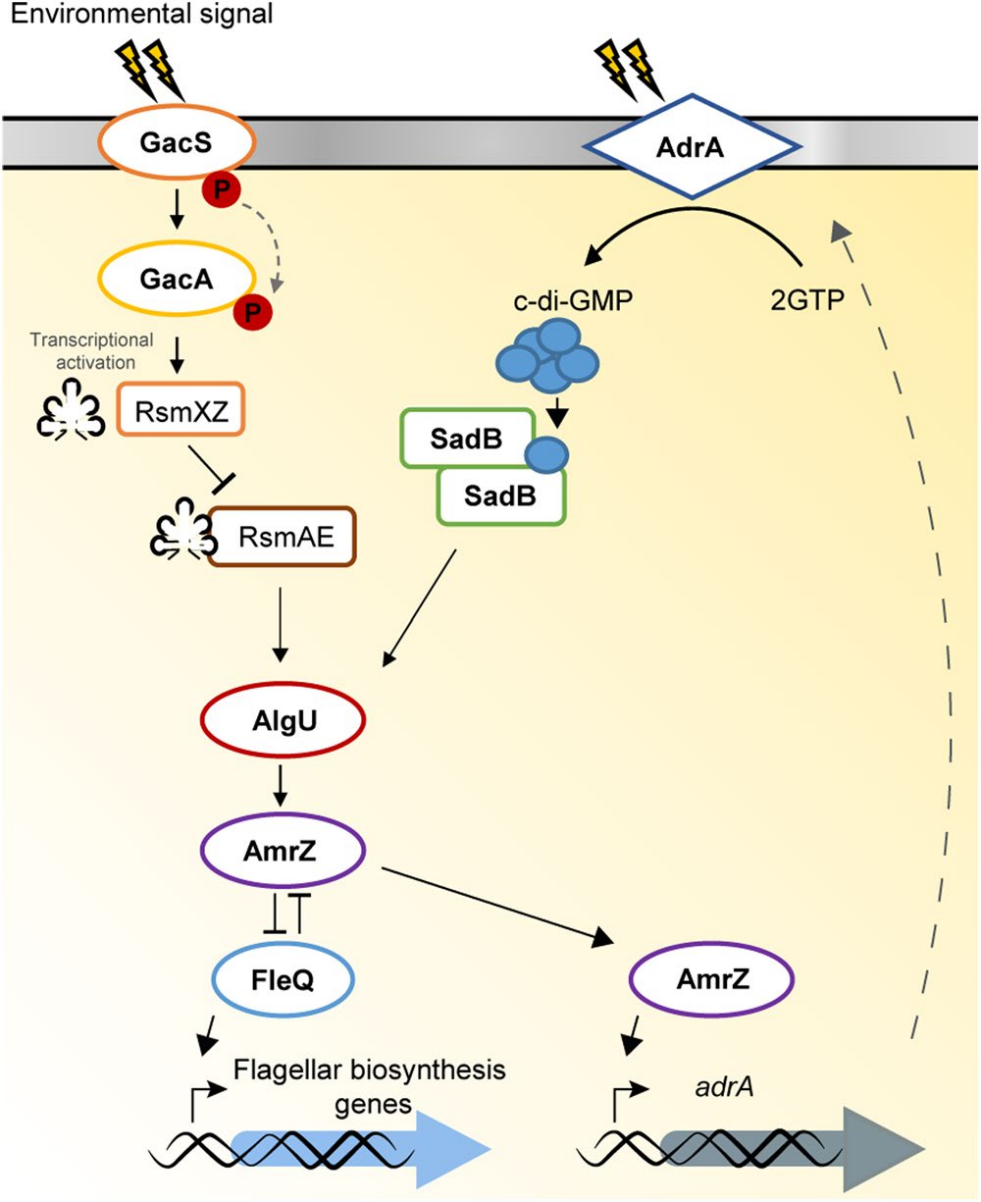
Hypothetical regulatory model for flagellar synthesis in *Pseudomonas fluorescens* F113. In this updated model, the transcriptional regulator AmrZ positively controls the expression of the gene encoding the transmembrane DGC AdrA. This DGC responds to unknown environmental signals and synthesizes the c-di-GMP that, when bound to SadB, is involved in the repression of the flagellar apparatus synthesis. SadB dimers bound to c-di-GMP activates the expression of the gene encoding the sigma factor AlgU, required for the expression of the gene encoding the global regulator AmrZ. From this point, the hub AmrZ/FleQ by means of a mutual transcriptional repression controls the expression of the flagellar biosynthesis genes. The Gac branch of the signaling pathway remains as described earlier^10^.

The results presented here allow us to modify the model for flagella synthesis regulation in *P. fluorescens* F113 presented earlier^10^. In the modified model (Fig. 5), two membrane bound signal transduction proteins, GacS and AdrA initiate the two branches of the pathway converging in AlgU. In the AdrA branch, c-di-GMP is produced by AdrA in response to unknown signals. This is sensed by SadB, which in turn activates *algU* transcription^10^, required for transcription of the *fleQ* transcriptional repressor AmrZ. AmrZ itself activates the transcription of the *adrA* gene^23^. Since the motility phenotype of the *sadB* mutant is stronger than the phenotype of the *adrA* mutant, it is likely that other factors act upon SadB, downstream of AdrA. On the other hand, it cannot be ruled out that c-di-GMP produced by AdrA could be sensed also by FleQ. However, the large difference in affinity for c-di-GMP between both proteins make this unlikely.

## Methods

### Strains and growth conditions

Bacterial strains and plasmids used in this study are listed in Supplementary Table 1. *P. fluorescens* F113 and derivatives were grown in Sucrose-Asparagine (SA) medium^43^ or Lysogeny Broth (LB) medium^44^ at 28 °C. *Escherichia coli* strains were grown in LB medium at 37 °C or 28 °C for protein overexpression. When required, the media were supplemented with agar (1.5%) and/or antibiotics. In the case of *Pseudomonas* strains: Rifampicin (Rif), 100 µg/mL; Tetracycline (Tet), 70 µg/mL; Kanamycin (Km), 50 µg/mL; Gentamicin (Gm), 3 µg/mL and Spectinomycin (Spc), 100 µg/mL. For *E. coli* strains the antibiotic concentrations were: Kanamycin (Km), 25 µg/mL; Tetracycline (Tet), 10 µg/mL; Gentamicin (Gm), 10 µg/mL and. Ampicillin (Amp), 100 µg/mL. For induction with pET*Nde*M-11^45^, β-D-thiogalactopyranoside (IPTG) was added to a final concentration of 0.5 mM.

### Molecular procedures

Routine molecular methods, including plasmid DNA extraction, cloning, restriction digests, electrophoresis, purification of DNA fragments and sequencing were performed in accordance with standard procedures.

Mutants were obtained by homologous recombination of an amplified internal fragment of the gene, cloned into the suicide vectors pK18*mobsacB*^46^, pG18*mob2*^47^ or pCR2.1 TOPO cloning (Life Technologies). Plasmids were mobilized into F113 by electroporation. Further information about mutant construction is shown in Supplementary Table 1. All mutants were checked by PCR and Southern blotting. Complementation of mutants and overexpression of genes was performed by expressing a wild-type copy of the gene in expression vector pBBR1MCS-5 (Supplementary Table 1)^48,49^.

Total RNA was extracted from *P. fluorescens* F113 and derivatives, grown in LB medium to exponential phase (OD_600_ = 0.8), following the instructions included in SV Total RNA Isolation System (Promega). The concentration and quality of RNA was determined using a Nanodrop spectrophotometer (Thermo Fisher Scientific). RNA integrity was confirmed on 0.8% (w/v) agarose gels. In addition, genomic DNA contamination in the samples was analysed by PCR. Complementary DNA (cDNA) synthesis by reverse transcription (RT-PCR) was performed using Superscript IV Reverse Transcriptase (Invitrogen) from 1 µg of extracted RNA. RT-qPCR analysis were carried out in triplicate for each gene using FastStart Universal SYBR Green Master Rox (Roche). Gene expression was normalized to the level of the endogenous housekeeping gene *rpoZ* and normalized to the wt reference sample following the 2^−ΔΔCt^ method^50^.

### Phenotypic analysis

Swimming motility assays were determined in SA (for *P. fluorescens*) or LB (for *E. coli*) plates containing 0.3% (w/v) purified agar as described before^28^. To induce expression of vector promoters, IPTG at 0.25 mM was used. Swimming haloes diameters were measured after 24 hours of incubation at 28 °C. Every assay was performed at least three times with three replicates in each experiment.

Biofilm formation assays were performed by a modified version of a previously described quantification method^51^. Briefly, overnight cultures grown in LB medium were adjusted to OD_600_ at 0.8 into fresh LB and statically incubated at 28 °C for 2 hours in 96-well-microlitre plates. Adhered cells were fixed with 99% methanol and stained with crystal violet. After washing and solubilization in acetic acid 33%, the absorbance of the eluted crystal violet was measured at OD_590_ on a Synergy HT multi-mode microplate reader (BioTek, Wilusky, VT, USA). Experiments were repeated three times with 16 technical replicates in each assay.

### c-di-GMP level determination

Fluorescence intensity emitted by *P. fluorescens* F113 and its *adrA* mutant harbouring the pCdrA::*gfp*C biosensor vector^52^ was visualized with a Leica M165 FC stereomicroscope employing a GFP filter set (Excitation/Emission 494/518 nm) with different exposure times. Pictures were collected with an exposure time elapsed to 50 milliseconds through Leica Application Suite software.

Indirect quantitative determination of intracellular c-di-GMP from wt and *adrA*^*−*^ strains harbouring pCdrA::*gfp*C vector was measured in 96-black well microplate assays, as described earlier^23^. Overnight cultures grown in LB medium were diluted to OD_600_ = 0.5 and fluorescence (excited at 485/20, emission at 528/20 nm) was measured in a Synergy HT multi-mode microplate reader (BioTek, Wilusky, VT, USA). Each experiment was performed in triplicate with 16 technical replicates.

### Protein Purification

*E. coli* BL21 (DE3) pLysS strain overexpressing SadB was grown overnight and then used to inoculate the overexpression cultures in a 1:100 dilution. These were grown at 37 °C to an OD_600_ of 0.4 in Terrific Broth (TB), before protein expression was induced overnight with 0.5 mM IPTG at 18 °C. Cells were then lysed by sonication and centrifuged at 15,000 g for one hour. SadB was purified from the supernatant by NTA-Ni chromatography using 1 mL HiTrap chelating HP columns (GE healthcare, life sciences). The columns were equilibrated with 10 volumes of washing buffer (20 mM HEPES pH 7.5, 250 mM NaCl, 2 mM MgCl_2_, and 2.5% (v/v) glycerol pH 6.8) and loaded with cell lysate. Following protein immobilization, the column was washed with 10 volumes of buffer containing 50 mM imidazole, before proteins were eluted using 500 mM imidazole buffer in a single step elution.

### Surface plasmon resonance (SPR)

The method was conducted as described by Trampari and coworkers^19^. SPR experiments were done at 25 °C with a Biacore T200 system (GE Healthcare) using a Streptavidin SA sensor chip (GE healthcare), which has four flow cells each containing SA pre-immobilized to a carboxymethylated dextran matrix. The chip was first washed three times with 1 M NaCl, 50 mM NaOH to remove any unconjugated streptavidin. 100 nM biotinylated c-di-GMP (BioLog B098) was immobilised on FC2 and FC4 of the streptavidin chip at a 50 RU immobilisation level with a flow rate of 5 μL/min. Flow cell one (FC1) and flow cell three (FC3) were kept blank to use for reference subtraction. Soluble SadB protein was prepared in SPR buffer (10 mM HEPES, 500 mM NaCl, 0.1% (v/v) Tween 20, 2 mM MgCl_2_, pH 6.8). Samples were injected with a flow rate of 5 μL/min over the reference and c-di-GMP cells for 60 seconds followed by buffer flow for 60 seconds. The chip was washed at the end of each cycle with 1 M NaCl. An increasing range of protein concentrations (78.125 nM, 156.25 nM, 312.5 nM, 625 nM, 1.25 µM, 2.5 µM, 5.0 µM, 10 μΜ) was used, with replicates for each protein concentration included as appropriate. All sensorgrams were analysed using Biacore T200 BiaEvaluation software version 1.0 (GE Healthcare). Data were then plotted using Microsoft Excel and GraphPad Prism 7.00 (GraphPad software, La Jolla, California, USA). The experiment was repeated three times independently.

### Biotinylated c-di-GMP pull-down experiment

For the overexpression of SadB 5 mL cultures were induced overnight at 18 °C using 0.5 mM IPTG. The cells were lysed by sonication and centrifuged for 30 minutes at 13,000 g. 45 μL of the soluble fraction was collected and mixed with biotinylated c-di-GMP (BioLog B098) at a final concentration of 30 μM. The mixture was then incubated overnight on a rotary wheel at 8 °C. For the stabilisation of any complex formation UV cross-linking was carried out using a UV Stratalinker (Stratagene) for 4 minutes on ice. 25 μL of streptavidin magnetic beads (Invitrogen) were then added into the mixture which was then incubated for 1 hour on a rotary wheel at 8 °C. A magnet was used to isolate the streptavidin magnetic beads and five washing steps were carried out using 200 μL of the protein washing buffer each time (20 mM HEPES pH 7.5, 250 mM NaCl, 2 mM MgCl_2_, and 2.5% (v/v) glycerol pH 6.8), to get rid of the non-specific c-di-GMP binding proteins. The washed streptavidin beads were resuspended in 15 μL protein washing buffer, 4x SDS loading dye was added and the samples were incubated at 95 °C for 10 minutes before loaded in a 12% SDS-PAGE protein gel. The gel was then developed using InstantBlue (Expedeon). GTP and c-di-GMP controls were added at 1 mM final concentration.

### Statistical analysis

GraphPad Prism 7.00 (GraphPad software, La Jolla, California, USA) was used for the statistical analysis. The comparison was done using one-way analysis of variance (ANOVA) followed by Tukey’s correction of multiple comparison test (p ≤ 0.05).

## Supplementary information

Supplementary information

## References

[1] DeFlaun MF, Marshall BM, Kulle EP, Levy SB (1994). Tn5 insertion mutants of *Pseudomonas fluorescens* defective in adhesion to soil and seeds. Appl Environ Microbiol.

[2] O’Toole GA, Kolter R (1998). Initiation of biofilm formation in *Pseudomonas fluorescens* WCS365 proceeds via multiple, convergent signalling pathways: a genetic analysis. Mol Microbiol.

[3] de Weert S (2002). Flagella-driven chemotaxis towards exudate components is an important trait for tomato root colonization by *Pseudomonas fluorescens*. Mol Plant Microbe Interact.

[4] Capdevila S, Martínez-Granero FM, Sánchez-Contreras M, Rivilla R, Martín M (2004). Analysis of *Pseudomonas fluorescens* F113 genes implicated in flagellar filament synthesis and their role in competitive root colonization. Microbiology.

[5] Muriel C, Jalvo B, Redondo-Nieto M, Rivilla R, Martín M (2015). Chemotactic motility of *Pseudomonas fluorescens* F113 under aerobic and denitrification conditions. PLoS One.

[6] Martínez-Granero F, Rivilla R, Martín M (2006). Rhizosphere selection of highly motile phenotypic variants of *Pseudomonas fluorescens* with enhanced competitive colonization ability. Appl Environ Microbiol.

[7] Barahona E (2010). Efficient rhizosphere colonization by *Pseudomonas fluorescens* f113 mutants unable to form biofilms on abiotic surfaces. Environ Microbiol.

[8] Arora SK, Ritchings BW, Almira EC, Lory S, Ramphal R (1997). A transcriptional activator, FleQ, regulates mucin adhesion and flagellar gene expression in *Pseudomonas aeruginosa* in a cascade manner. J Bacteriol.

[9] Dasgupta N (2003). A four-tiered transcriptional regulatory circuit controls flagellar biogenesis in Pseudomonas aeruginosa. Molecular microbiology.

[10] Martínez-Granero F (2012). The Gac-Rsm and SadB signal transduction pathways converge on AlgU to downregulate motility in *Pseudomonas fluorescens*. PLoS One.

[11] Martínez-Granero F, Redondo-Nieto M, Vesga P, Martín M, Rivilla R (2014). AmrZ is a global transcriptional regulator implicated in iron uptake and environmental adaption in *P. fluorescens* F113. BMC Genomics.

[12] Jones CJ (2014). ChIP-Seq and RNA-Seq reveal an AmrZ-mediated mechanism for cyclic di-GMP synthesis and biofilm development by *Pseudomonas aeruginosa*. PLoS Pathog.

[13] Tart AH, Blanks MJ, Wozniak DJ (2006). The AlgT-dependent transcriptional regulator AmrZ (AlgZ) inhibits flagellum biosynthesis in mucoid, nonmotile *Pseudomonas aeruginosa* cystic fibrosis isolates. J Bacteriol.

[14] Hengge R (2009). Principles of c-di-GMP signalling in bacteria. Nat Rev Microbiol.

[15] Simm R (2004). domains inversely regulate cyclic di-GMP levels and transition from sessility to motility. Mol Microbiol.

[16] Tal R (1998). Three *cdg* operons control cellular turnover of cyclic di-GMP in *Acetobacter xylinum*: genetic organization and occurrence of conserved domains in isoenzymes. J Bacteriol.

[17] Ausmees N (2001). Genetic data indicate that proteins containing the GGDEF domain possess diguanylate cyclase activity. FEMS Microbiol Lett.

[18] Aravind L, Koonin EV (1998). The HD domain defines a new superfamily of metal-dependent phosphohydrolases. Trends in biochemical sciences.

[19] Trampari E (2015). Bacterial rotary export ATPases are allosterically regulated by the nucleotide second messenger cyclic-di-GMP. J Biol Chem.

[20] Martínez-Granero F (2014). Identification of *flgZ* as a flagellar gene encoding a PilZ domain protein that regulates swimming motility and biofilm formation in *Pseudomonas*. PLoS One.

[21] Baker AE (2016). PilZ domain protein FlgZ mediates cyclic di-GMP-dependent swarming motility control in *Pseudomonas aeruginosa*. J Bacteriol.

[22] Petrova OE, Cherny KE, Sauer K (2014). The *Pseudomonas aeruginosa* diguanylate cyclase GcbA, a homolog of *P. fluorescens* GcbA, promotes initial attachment to surfaces, but not biofilm formation, via regulation of motility. J Bacteriol.

[23] Muriel C (2018). AmrZ is a major determinant of c-di-GMP levels in *Pseudomonas fluorescens* F113. Sci Rep.

[24] Hickman JW, Harwood CS (2008). Identification of FleQ from *Pseudomonas aeruginosa* as a c-di-GMP-responsive transcription factor. Mol Microbiol.

[25] Baraquet C, Harwood CS (2013). Cyclic diguanosine monophosphate represses bacterial flagella synthesis by interacting with the Walker A motif of the enhancer-binding protein FleQ. Proc Natl Acad Sci USA.

[26] Finn RD (2015). HMMER web server: 2015 update. Nucleic Acids Res.

[27] Nikolskaya AN, Mulkidjanian AY, Beech IB, Galperin MY (2003). MASE1 and MASE2: two novel integral membrane sensory domains. J Mol Microbiol Biotechnol.

[28] Navazo A (2009). Three independent signalling pathways repress motility in *Pseudomonas fluorescens* F113. Microb Biotechnol.

[29] Zhang H (2000). Crystal structure of YbaK protein from *Haemophilus influenzae* (HI1434) at 1.8 A resolution: functional implications. Proteins.

[30] Merritt JH, Brothers KM, Kuchma SL, O’Toole GA (2007). SadC reciprocally influences biofilm formation and swarming motility via modulation of exopolysaccharide production and flagellar function. J Bacteriol.

[31] Blanco-Romero E (2018). Genome-wide analysis of the FleQ direct regulon in *Pseudomonas fluorescens* F113 and *Pseudomonas putida* KT2440. Sci Rep.

[32] Baraquet C, Murakami K, Parsek MR, Harwood CS (2012). The FleQ protein from *Pseudomonas aeruginosa* functions as both a repressor and an activator to control gene expression from the *pel* operon promoter in response to c-di-GMP. Nucleic Acids Res.

[33] Römling U, Rohde M, Olsén A, Normark S, Reinköster J (2000). AgfD, the checkpoint of multicellular and aggregative behaviour in *Salmonella typhimurium* regulates at least two independent pathways. Mol Microbiol.

[34] Topal H (2012). Crystal structure and regulation mechanisms of the CyaB adenylyl cyclase from the human pathogen *Pseudomonas aeruginosa*. J Mol Biol.

[35] Cowles KN, Willis DK, Engel TN, Jones JB, Barak JD (2016). Diguanylate xyclases AdrA and STM1987 regulate *Salmonella enterica* exopolysaccharide production during plant colonization in an environment-dependent manner. Appl Environ Microbiol.

[36] Garrido-Sanz D (2016). Genomic and genetic diversity within the *Pseudomonas fluorescens* complex. PLoS One.

[37] Garrido-Sanz D (2017). Classification of isolates from the *Pseudomonas fluorescens* complex into phylogenomic groups based in group-specific markers. Front Microbiol.

[38] Caiazza NC, Shanks RM, O’Toole GA (2005). Rhamnolipids modulate swarming motility patterns of *Pseudomonas aeruginosa*. J Bacteriol.

[39] Galperin MY (2004). Bacterial signal transduction network in a genomic perspective. Environ Microbiol.

[40] Lee HM (2016). Characterization of genes encoding proteins containing HD-related output domain in *Xanthomonas campestri*s pv. campestris. Antonie Van Leeuwenhoek.

[41] Liu YF (2013). GsmR, a response regulator with an HD-related output domain in *Xanthomonas campestris*, is positively controlled by Clp and is involved in the expression of genes responsible for flagellum synthesis. FEBS J.

[42] Caiazza NC, O’Toole GA (2004). SadB is required for the transition from reversible to irreversible attachment during biofilm formation by *Pseudomonas aeruginosa* PA14. J Bacteriol.

[43] Scher FM, Baker R (1982). Effect of *Pseudomonas putida* and a synthetic iron chelator on induction of soil suppressiveness to *Fusarium* wilt pathogens. Phytopathology.

[44] Bertani G (1951). A method for detection of mutations, using streptomycin dependence in *Escherichia coli*. Genetics.

[45] Little R, Salinas P, Slavny P, Clarke TA, Dixon R (2011). Substitutions in the redox-sensing PAS domain of the NifL regulatory protein define an inter-subunit pathway for redox signal transmission. Mol Microbiol.

[46] Schäfer A (1994). Small mobilizable multi-purpose cloning vectors derived from the *Escherichia coli* plasmids pK18 and pK19: selection of defined deletions in the chromosome of *Corynebacterium glutamicum*. Gene.

[47] Kirchner O, Tauch A (2003). Tools for genetic engineering in the amino acid-producing bacterium *Corynebacterium glutamicum*. J Biotechnol.

[48] Kovach ME (1995). Four new derivatives of the broad-host-range cloning vector pBBR1MCS, carrying different antibiotic-resistance cassettes. Gene.

[49] Heeb S, Blumer C, Haas D (2002). Regulatory RNA as mediator in GacA/RsmA-dependent global control of exoproduct formation in *Pseudomonas fluorescens* CHA0. J Bacteriol.

[50] Livak KJ, Schmittgen TD (2001). Analysis of relative gene expression data using real-time quantitative PCR and the 2(-Delta Delta C(T)) Method. Methods.

[51] Peeters E, Nelis HJ, Coenye T (2008). Comparison of multiple methods for quantification of microbial biofilms grown in microtiter plates. J Microbiol Methods.

[52] Rybtke MT (2012). Fluorescence-based reporter for gauging cyclic di-GMP levels in *Pseudomonas aeruginosa*. Appl Environ Microbiol.

